# Study on JV Virus in Patients with Colon Cancer Type Adenocarcinoma

**DOI:** 10.31557/APJCP.2019.20.4.1147

**Published:** 2019

**Authors:** Azadeh Haghi Navand, Ali Teimoori, Manoochehr Makvandi, Nilofar Nisi, Seyed Saeid Seyedian, Nastarn Ranjbari, Kambiz Ahmadi Angali, Hadis Keyani, Maryam Tabasi, Keyvan Pourjabari

**Affiliations:** 1 *Infectious and Tropical Diseases Research Center Health Research Institute, *; 2 *Virology Department, School of Medicine,*; 3 *Alimentary Tract Research Center, *; 4 *Department of Pathology, Imam Khomeini Hospital,*; 5 *Department of Biostatistic, School of Health, Ahvaz Jundishapur University of Medical Sciences, Ahvaz, Iran. *

**Keywords:** JC virus, colorectal adenocarcinoma, polymerase chine reaction

## Abstract

**Introduction:**

Colorectal cancer is the most repetitious malignancies with high mortality worldwide. JC virus (JCV) is ubiquitous Polyomavirus, with seroprevalence rates ranging from 70% to 90% in adult population. Recently the role of JCV have been reported in many malignant tumors worldwide. The association of JCV was reported in patients with colon and rectum cancers. Thus this study was conducted to evaluate the association of JCV DNA in patients with colon cancer type Adenocarcinoma.

**Material and Methods:**

A total of 120 formalin-fixed paraffin-embedded tissue blocks samples were collected including 20/40(50%) males, 20/40(50%) females patients with Colorectal Cancer(CRC), and 80 (50% males, 50% females) patients with benign tumor as a control. DNA was extracted for all the samples. Nested PCR was carried out for detection of Vp1/T-Ag junction genome in JCV genome by Nested-PCR assay. Randomly, PCR products of 6 samples were sequenced to analysis the partial JCV DNA. The phylogeny tree was constructed to determine homology identity with other JCV.

**Results:**

4/40(10%) samples of test group and 10/80 (12.5%) of control samples were positive for JCV DNA (P= 0.69). Out of 4 samples positive for JC DNA, 3(7.5%) were males and 1(2.4%) female (P=0.29). The frequency of JCV DNA in age group> 50 years was 4/32(10%), while in age group <50 years was 0/8 (0%) (p= 0.29).

**Conclusion:**

prevalence of JCV DNA was among 10% patients with CRC and 12.5% benign tumors (p=0.69). The distribution of JCV DNA was among 7.5% male and 2.5% female (p= 0.29). The frequency of JCV DNA was among 10% cases of age group >50 years and 0% of age group <50 years (P= 0.29). The subsequent T-Ag protein expression might explain the increased risk of colorectal cancer and requires further investigation.

## Introduction

Colorectal cancer (CRC) is the most recurrent malignancy, and third leading cancer with high mortality among men and women worldwide (World Cancer Research Fund, 2018). Most colon cancers are classiﬁed as adenocarcinomas and subdivided into low-grade and high-grade (Ugo et al., 2018). The pathogenesis of this tumor is not well known. Predisposing factors include Crohn’s disease, celiac disease, hereditary genetic syndromes and dietary factors (Zaaimi et al., 2015). JCV is a polyomavirus that infects most humans population without clinical sign and symptoms worldwide. JCV causes the lethal demyelinating disease, progressive multifocal leukoencephalopathy (Padgett et al., 1971). About 90% of the adult population carries antibodies to the virus, and it seems that in most people, the virus remains latent (Knowles, 2006). High concentrations of JCV have been observed in urban sewage worldwide, therefore, it is suspected that the contaminated water is a typical route of JCV infection (Dias et al., 2018).

JCV is detected in the normal gastrointestinal tract and its complicity in colorectal cancer in humans (Toumi et al., 2017; Sinagra et al., 2014). JCV encodes three proteins that are the structural capsid proteins: VP1, VP2, and VP3 (Zheng et al., 2005). JCV also encodes the T antigen proteins (i.e., Large T and multiple small t splice variants) and the agnoprotein which is involved in the assembly of viral particles. T-Ag has several splice sites producing truncated proteins that are involved in regulating cell proliferation and viral transmission (Frisque, 2001). Based on nucleotide difference in VP1 region of the JCV, 8 genotypes and 14 subtypes was recognized (Caiqin et al., 2018). JCV genoptypes 1, 2, and 4 are distributed in Europe. Subtypes 3 and 6 are found in Africa, subtype 8 is found in Papua New Guinea and the Pacific Islands, and subtypes 2, 4, and 7 are found in Asia (Zanotta et al., 2013). 

T-Ag is capable of disrupting the function of tumor suppressor proteins p53 and members of the pRB family, which probably contributes to carcinogenesis in the animal models (Qian et al., 2000; Desjardins et al., 1997). The nucleic acid and T-Ag of JCV has been found in broad spectrum of human tumors such as esophageal carcinoma, colorectal cancer, anal carcinoma and gastric cancers (Del et al, 2005; Coelho et al., 2010; Link et al., 2009; Mou et al., 2012; Ramamoorthy et al.,2011; Shin et al., 2006). Based on experimental models, the human polyomaviruses JC (JCPyV) and BK (BKPyV) have been recently categorized by the International Agency for Research in Cancer as “possible carcinogens (Bouvard et al., 2012).

Studies on association of JCV and colorectal adenocarcinoma in Iran is limited, therefore, this study was conducted to evaluate the rate of JCV in patients with CRC in Ahvaz city. Ahvaz city is capital of khozestan province, located in the south west region of Iran.

*Ethic Consent*

The project was approved with approval number OG93141 by ethic committee of Ahvaz Jundishapur university of Medical Sciences, Ahvaz, Iran.

## Materials and Methods

*Sample preparation*

A total of 120 formalin-fixed paraffin-embedded tissue blocks samples were collected from 40 (50% male and 50% female) patients with CRC and 80 healthy control groups(40 male and 40 female) from Golestan and Imam Khomeyni hospitals in Ahvaz city, during 2004 to 2015. The sections of 5 microns thickness were prepared from each sample and stored at 4°C until DNA extraction.

Deparaffinization: Deparaffinization was done by xylene and ethanol (Germany, Merk). Initially, all the specimens were placed in microtubes then xylene was added and kept at 45^o^C for 15 min followed by centrifuge at 14,000 rpm. This stage was repeated again. The supernatant was discarded and 1ml absolute ethanol was added to precipitate and stored at the room temperature for 10 min and centrifuged again at 14,000 rpm for 1 minute. The supernatant was discarded. This process was repeated by adding 70% ethanol, followed the same condition. Finally supernatant was discarded and all microtubes were placed at 65^o^C for 5 min to vaporize the ethanol residue and the pellet was used in DNA extraction (Habibian et al., 2013 ).

*DNA extraction*

High pure PCR template preparation kit (Roche, Germany, code No: 11796828001) was applied for the extraction of DNA, according to the manufacturer’s instruction. The extracted DNA was stored at -70°C until PCR amplification.

*PCR amplification*

All the extracted DNA samples were initially subjected to PCR with consensus primers PCO3/PCO4 (β-globin) to confirm the quality of the extracted DNA (used as an internal control). The following primers (PCO3: 5 ´ACA CAACTGTGTTCACTAGC/PCO4: 5́ CAACTTCAT CCACGTTCACC with PCR product of 110 bp (Shahab et al., 2015). For detection of JCV DNA by Nested PCR, the following primers and thermal programs were used. The specific primer P1 and P2 was used for amplification of JCV Vp1/T-Ag junction region of JCV (Tsuyoshi et al., 1995). The PCR reaction mixture consisted of PCR, MgCl_2_ (25mM) 2µl, dNTP (10mM) 0.5 µl, primers each 1 µl, taq polymerase(5unit) 0.3 µl, D/W 12.5 µl, and template 7µl. Based on the complete genome of JCV, accession NO; J02226.1. P1(VP1) -forward primer (outer) (2107-2127) 5’-TTTTGGGACACTAACAGGAGG-3’ and P2- Reverse primers (outer) (VP1- Large T Ag)(2743-2762) 5’-AGCAGAAGACTCTGGACATG-3’ was used with thermal condition as follow: initial denaturation at 94^o^C for 5 minutes, 35 cycles at 94^o^C for 1 min, 52^o^C for 1 min,72^o^C for 1 min, and a final extension 72^o^C for 10 min. 5µl of first round PCR products, was used for second round nested PCR. The following primers the inner forward primer (VP1) (2150-2172) 5’- CATATAACAAACACTGCCACAAC-3’ and inner reverse primer (2696-2716) (VP1- Large T Ag) 5’- TGCTTTTCCCAGGTCTCAGAA -3 with the same PCR reaction mixture as described in the first round, was subjected to thermocyler (Teqlab, Germany) with thermal program as follow: initial denaturation at 94^o^C for 5 min followed by 35 cycles of denaturation at 94^o^C for 1 min, annealing at 49^o^C for 1 min, and extension at 72^o^C for 1 min and the final extension step at 72^o^C for 10 min. All reactions performed in duplicate and along with the negative (distilled water) and positive controls. 

*Gel electrophoresis*

**Figure 1:**
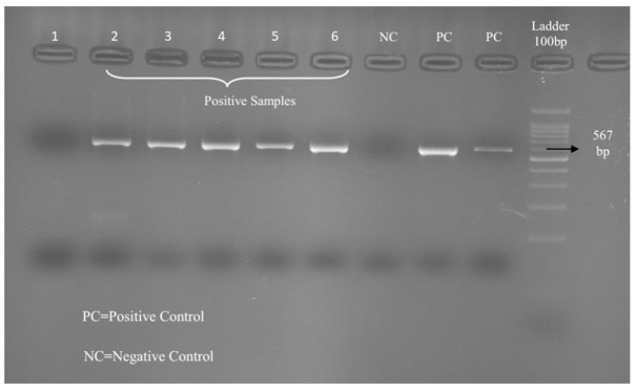
Results of JCV PCR in Patients with CRC. Line 1, Negative sample; Line 2-6, Positive samples; NC, Negative Control; PC, Positive sample; Molecular Marker (100bp size)

The PCR products were separated on a 2% agarose gel and developed by Safe Stain under voltage at 100V. The result was seen under ultra violet in transilluminator. The first round PCR product was 656bp and second PCR products 567bp (Figure 1). The sizes of bands were compared with 100bp Ladder (Fermentas) which, was placed on the well as an indicator.

*Sequencing*

To confirm the results of PCR and to determine JCV DNA randomly 6 positive PCR products were selected and sequenced (Bioneer company, South Korea). The sequences were blasted using available databases https://www.ncbi.nlm.nih.gov. 

*Statistical analysis*

The obtained results were analyzed by the version 17 of SPSS software and the role of age and sex on positive cases were surveyed by the Fisher`s exact and Chi square test.

## Results

In this survey 4/40(10%) samples of CRC and 10/80 (12.5%) of control samples were positive for JCV DNA (P= 0.69). The frequency of JCV was among the age group >50 years, 4/32 (10%) compared to <50 years , 0/8 (p=0.29). 3/4 (7.5%) of male patients with CRC showed positive for JCV DNA while 1/4 (2.5%) of female patients with CRC exhibited positive for JCV DNA (p=0.29). The rate of JCV in pathogenic stage, grade I, II was 0/4 (0%) while in grade III, IV 4(10%) was not significant (p=0.48). The distribution of JCV DNA in differentiation stage was WDAC 1 (2.5%) , MDAC 3(7.5%) and PDAC (0%) (p= 0.53). 

Figure 1, shows the results of positive and negative samples by PCR. Table 1 shows the profile of patients with CRC positive and negative for JCV DNA.

**Figure 2:**
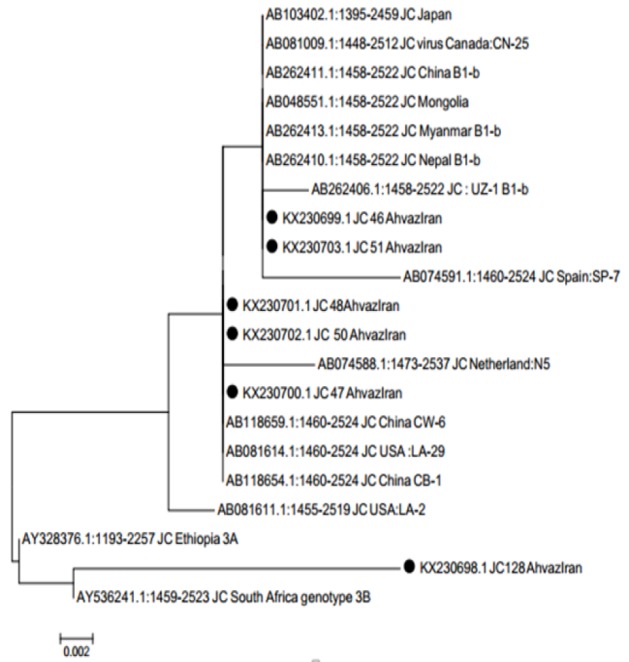
Phylogeny Tree was Constructed by Maximum Likelihood Method for JCV Vp1/T-Ag Junction Region of the Isolated JC virus Genomes with Accession Number KX230698- KX230703 Labeled by the Black Solid Circle. TThey were compared with different JCV Vp1/TAg junction isolated from different regions of the world with accession numbers retrieved from Gen Bank

The results of 6 sequences of the JCV Vp1/T-Ag junction region of the isolated JCV were deposited at GenBank under the accession numbers KX230698- KX230703. The MEGA6 and Maximum Likelihood method were used for phylogenetic analysis (Figure 2).

## Discussion

Several factors including status of host genetic, immunodeficiency, geographically endemic patterns of JC virus infection, co-infection of JC virus and papillomavirus may lead to adenocarcinoma tumors (Shin et al., 2006; Yamaoka et al., 2009). CRC is one of dominant cause of death among man and women worldwide . High prevalence of 82% and 90% of JC virus associated with colorectal carcinoma have been reported in Portugal and USA respectively (Coelho et al., 2013; Shalaka et al., 2014). In the present study 10% of CRC of patients were positive for JCV DNA and was in agreement with results reported by Mou et al., (2012) in China. Sarvari et al., (2018) have reported low prevalence of 1.42% JCV DNA in patients with CRC, in Shiraz city, Iran, which was lower than our finding. In the present study the rate of JCV DNA was among the male (7.5%) and female (2.5%) patients (p=0.29%). Shalaka et al., (2014), depicted that the rate of JCV in male patients with CRC is higher than female. In the present study the frequency of JCV DNA among age group >50 years (10%), and age group <50 years (0%) (P= 0.29). Mou et al., (2012) have reported in China the frequency of JC DNA among the age groups was not found significant. 

Investigations have revealed that almost half of gastric cancers samples were positive for JCV (Yamaoka et al., 2009; Shin et al., 2006). The concomitant JCV and human papillomavirus (HPV) have been reported. Twenty two anal cancers were tested by PCR for JCV and all were positive, while concomitantly 13 of the cases were positive for HPV (Ramamoorthy et al., 2011). These data demonstrate the presence of JCV throughout cancers of the digestive tract and suggest the possibility that JCV initiates cancers in these tissues. 

it has been hypothesized that JC virus T-Ag could mediate metastasis in CRC cells through increased migration and invasion (Link et al., 2009)

In our present study the high frequency of 10% JCV DNA have been found among the CRC specimens with grade III, IV. Mou et al., (2012) have detected the JCV DNA in CRC specimens with grade I, II, III, IV.

Several bacteria have been identified and implicated in the development of CRC. These include: Streptococcus bovis (Knudson, 2001), Helicobacter pylori (Moss et al., 1995), and Fusobacterium (Castellarin et al., 2012). Several viruses were suggested to be the risk factors for CRC. Among them, John Cunningham virus (JC virus), BK virus, Human Cytomegalovirus (CMV), Human papilloma viruses (HPV: particularly type 16 and 18) have the largest number of report (Vlado et al., 2013)

In the present study about 36/40 (90%) samples showed negative for JC DNA. The role of mentioned bacteria and viruses have not investigated but requires further investigation. 

**Table 1:** Shows the Number of Positive and Negative JCV in Tumors

Category	JC virus positive	JC virus negative	p Value
Ages			
>50	4 (10%)	28 (70%)	0.29
<50	-	8 (20%)	
Gender			
Male	3 (15%)	17 (85%)	0.29
Female	1 (5%)	19 (95%)	
Colocteral Adenocarcinoma	4 (10%)	36 (90%)	0.69
Benign tumor	10 (12.5%)	70 (87.5%)	
Pathogenic stage			
Grade I, II	-	4 (10%)	0.48
Grade III, IV	4 (10%)	32 (80%)	
Differentiation stage			
WDAC	1 (2.5%)	5 (12.5%)	0.53
MDAC	3 (7.5%)	23 (57.5%)	
PDAC	-	8 (20%)	

Table 1 shows the distributions of JC Virus DNA was among the age groups(p=0.29%), in gender (p=0.29%) in CRC and benign tumor (p=0.69), in pathology grade III, IV with histology grade I and II (p=0.48) and in Differentiation stage was not found significant (p=0.53%).

The isolated JC virus from Ahvaz, with accession number KX230698- KX230703 showed 99% homology identity with different JC virus isolated from Japan, China, Mongolia, Uzbekistan, Spain, USA and Netherland. Scale Bar: 0.0002

Recent investigations revealed that, Infected patients with JC virus in immunodeficiency status may results in progress to PML, thus, treatment of patients infected with JC virus by rituximab natalizumab and efalizumab lead to progressive multifocal leukoencephalopathy (David et al., 2011; McGuigan et al., 2016; Schwab et al., 2012). Therefore, it is suggested that the urine of patients with CRC cancer or patients with autoimmune diseases should be screened for JC virus DNA before chemotherapy treatment or by immunomodulatory drugs (rituximab and natalizumab) therapy.

In summary, prevalence of JCV DNA was among 10% patients with CRC and 12.5% benign tumors (p=0.69). The distribution of JCV DNA was among 7.5% male and 2.5% female (p= 0.29). The frequency of JCV DNA was among 10% cases of age group >50 years and 0% of age group <50 years (P= 0.29). 

The subsequent T-Ag protein expression might explain the increased risk of colorectal cancer and requires further investigation. Recent investigations revealed that, Infected patients with JC virus in immunodeficiency status may results in progress to PML, thus, treatment of patients infected with JC virus by rituximab natalizumab and efalizumab lead to progressive multifocal leukoencephalopathy. Therefore, it is suggested that urine of patients with CRC cancer or patients with autoimmune diseases should be screened for JC virus DNA before chemotherapy treatment or by immunomodulatory drugs ( rituximab and natalizumab) therapy.
